# “Good” fats, bad news: HDL-delivered vitamin E shields tumors from ferroptosis

**DOI:** 10.1038/s41392-025-02373-x

**Published:** 2025-09-02

**Authors:** Dong Wook Choi, Eun-Woo Lee

**Affiliations:** 1 Department of Biotechnology, College of Life Sciences and Biotechnology, Korea University, Seoul, Republic of Korea; 2 Metabolic Regulation Research Center, Korea Research Institute of Bioscience and Biotechnology (KRIBB), Daejeon, Korea; 3 School of Pharmacy, Sungkyunkwan University, Suwon, Korea

**Keywords:** Cancer metabolism, Cell biology

In a recent study published in *Nature*, Calhoon et al.^1^ reported that glycosaminoglycan (GAG)-mediated uptake of lipoproteins protects cancer cells from ferroptosis by delivering α-tocopherol, the most abundant form of vitamin E. This discovery offers potential therapeutic opportunities to either target GAG-dependent lipid uptake or modulate dietary vitamin E to enhance ferroptosis-based cancer therapies.

Dynamic reprogramming of cellular metabolic pathways is a hallmark of cancer. Among these, cancer cells actively exploit a diverse range of lipid species, not only as essential biosynthetic precursors (e.g., membrane lipids) and metabolic fuels, but also for maintaining cellular redox homeostasis. While tumors benefit from such metabolic rewiring, their unique metabolic snapshot offers attractive opportunities for cancer-targeted approaches. One notable case is ferroptosis, an iron-dependent, regulated cell death triggered by lipid peroxidation, particularly within polyunsaturated fatty acid (PUFA)-enriched phospholipids, whose synthesis relies on exogenous essential fatty acids.^2,3^ Although cancer cells satisfy lipid demands via both de novo synthesis and extracellular lipid uptake, how these complex metabolic inputs impact ferroptosis sensitivity remains elusive.

To investigate this, Calhoon et al. focused on lipoproteins, key carriers of circulating lipids that facilitate lipid uptake through receptor-mediated mechanisms. A pooled CRISPR screen targeting 200 metabolism-related genes in HeLa cells revealed that glutathione peroxidase 4 (*GPX4*), a crucial ferroptosis suppressor, was selectively essential in lipoprotein-depleted environments, leading to the hypothesis that lipoproteins may confer protection against ferroptosis. Supporting this idea, supplementation with either high-density lipoprotein (HDL) or low-density lipoprotein (LDL) restored the viability of GPX4-deficient cells, albeit with HDL offering greater protection. They also showed that the protective capacity is independent of cholesterol content. To determine whether this effect is attributed to direct modulation of lipid peroxidation, the authors employed the fluorescence-enabled inhibited autoxidation (FENIX) assay, which quantifies liposomal membrane peroxidation. Notably, lipoproteins in which native core lipids were replaced with tristearin, a neutral triglyceride composed of three stearate (C18:0) chains, displayed an ablated activity, indicating native core lipids as an essential component for the lipid peroxidation-suppressive activity of lipoproteins.

**Fig. 1:**
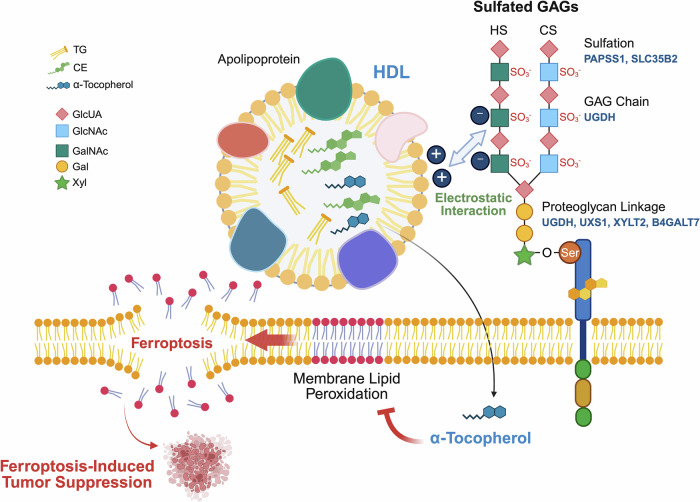
Sulfated GAG-mediated HDL uptake delivers α-tocopherol and suppresses ferroptosis in cancer cells. HDL’s high α-tocopherol content directly inhibits lipid peroxidation both in vitro and in vivo, thereby suppressing ferroptosis and fostering tumor growth. A CRISPR screen identified genes essential for HDL uptake and ferroptosis suppression, including *UGDH*, which is involved in GAG chain synthesis, and *UGDH*, *UXS1*, *XYLT2*, and *B4GALT7*, which mediate proteoglycan linker assembly. These genes contribute to the biosynthesis of sulfated GAG chains such as chondroitin sulfate (CS) and heparan sulfate (HS), together with sulfation-related genes such as *PAPSS1* and *SLC35B2*. All genes highlighted in blue were identified from the CRISPR screen. The positively charged apolipoproteins on HDL interact with the negatively charged sulfate groups on GAGs, triggering HDL uptake via an unknown mechanism. B4GALT7 beta-1,4-galactosyltransferase 7, Gal galactose, GalNAc N-acetylgalactosamine, GAG glycosaminoglycan, GlcNAc N-acetylglucosamine, GlcUA glucuronic acid, HDL high-density lipoprotein, PAPSS1 3′-phosphoadenosine 5′-phosphosulfate synthase 1, SLC35B2 solute carrier family 35 member B2, UGDH UDP-glucose 6-dehydrogenase, UXS1 UDP-glucuronic acid decarboxylase 1, XYLT2 xylosyltransferase 2. Figure was created with BioRender.com (https://BioRender.com/wp21cb5)

The authors next examined candidate lipid metabolites that might exert HDL’s protective effect, focusing on those known for their anti-ferroptotic properties. These include α-tocopherol (vitamin E), vitamin K2, and coenzyme Q10 (CoQ10), which act as radical-trapping antioxidants, and monounsaturated fatty acids (MUFAs), which reduce membrane susceptibility to peroxidation by competing with PUFAs. To this end, they first disrupted two key enzymes: long-chain-fatty-acid–CoA ligase 3 (*ACSL3*), which facilitates MUFA incorporation into membrane phospholipids, and apoptosis-inducing factor mitochondria-associated 2 (*AIFM2*, also known as ferroptosis suppressor protein 1, FSP1), which regenerates the antioxidant forms of CoQ10 and vitamin K2. Interestingly, HDL retained its lipid peroxidation- and ferroptosis-suppressive activity in both ACSL3- and FSP1-deficient cells, ruling out those pathways. Notably, untargeted lipidomics identified that α-tocopherol, the most abundant vitamin E species within HDL, is responsible for HDL’s superior anti-ferroptotic activity (Fig. 1). Plasma from vitamin E-deficient mice, but not mice fed a control diet, failed to protect GPX4-deficient cancer cells from ferroptosis, and in tumor xenograft models, cancer cells implanted into vitamin E-deficient mice exhibited slower growth than those in control animals. These results suggest that dietary vitamin E promotes tumor growth by protecting cells from ferroptotic stress.

Given that dietary vitamin E restriction has limited therapeutic potential in cancers, the authors sought to identify molecular mechanisms underlying vitamin E uptake by cancer cells. To this end, the authors performed two independent CRISPR screens in Karpas299 cells, a lymphoma cell line displaying high lipoprotein uptake: one aimed to identify genes required for resistance to ML210-induced ferroptosis, while the other was conducted to uncover genes involved in lipoprotein uptake. Both screens converged on genes involved in the biosynthesis of sulfated GAGs (cell-surface sulfated glycosaminoglycans), which are long, linear polysaccharides (Fig. 1). Loss of these genes impaired the synthesis of heparan sulfate (HS) and chondroitin sulfate (CS), the two most abundant sulfated GAGs, reduced lipoprotein uptake, and sensitized diverse cancer cell lines to ferroptosis. Furthermore, enzymatic degradation of sulfated GAGs with heparinases and chondroitinase diminished HDL and LDL internalization and restored ferroptosis under lipoprotein-supplemented conditions. Therefore, the authors suggest that sulfated GAGs facilitate electrostatic interactions with apolipoproteins, thereby enhancing cell-surface binding and endocytosis of lipoproteins although the precise mechanism remains unclear (Fig. 1). In contrast, deletion of canonical lipoprotein receptors such as scavenger receptor class B1 (*SCARB1*) or LDL receptor-related protein 8 (*LRP8*) had marginal effects, underscoring the dominant role of GAG-dependent uptake.

Further investigation into human clear cell renal cell carcinomas (ccRCCs), a lipid-rich malignancy, revealed significantly elevated CS levels and increased lipoprotein-derived α-tocopherol compared with normal kidney tissues. Consistent with this, patient-derived xenograft (PDX) models with genetic depletion or enzymatic degradation of GAGs exhibited reduced LDL uptake and impaired tumor growth, reinforcing GAG biosynthesis as a critical determinant of tumor growth by mitigating lipid oxidative stress. Thus, GAGs provide a key anti-ferroptotic mechanism, offering a potential therapeutic target for unleashing ferroptosis-based cancer therapies.

While antioxidants such as vitamin E were once considered protective against cancer, the Selenium and Vitamin E Cancer Prevention Trial (SELECT) paradoxically revealed an increased risk of prostate cancer,^4^ leading to an emerging view that ferroptosis inhibition by dietary antioxidants may underlie such outcomes. In this emerging perspective, the present study provides mechanistic insight into how lipoprotein-derived vitamin E protects cancer cells from ferroptosis. Collectively, this study highlights α-tocopherol as a key lipoprotein-derived antioxidant that protects cancer cells from ferroptosis via GAG-mediated uptake. While α-tocopherol is a key component, lipoproteins may also deliver additional lipid species involved in ferroptotic vulnerability given their diverse composition. Thus, the broader impact of lipoprotein uptake on lipidomic remodeling and ferroptosis sensitivity in cancer warrants further investigation. While targeting extracellular lipid uptake to sensitize cancer cells to ferroptosis induction offers an attractive therapeutic strategy, it remains unclear how the identified mechanisms of sulfated GAGs and lipoprotein uptake influence immune cells within the tumor microenvironment, which are known to significantly impact cancer growth and the efficacy of targeted therapy.^5^ Therefore, a deeper understanding of how sulfated GAGs and lipoprotein uptake affect ferroptosis across different cell types in the tumor milieu may reveal new therapeutic opportunities.
